# Implementing the WHO caregivers skills training program with caregivers of autistic children *via* telehealth in rural communities

**DOI:** 10.3389/fpsyt.2022.909947

**Published:** 2022-08-29

**Authors:** Cecilia Montiel-Nava, Megan Tregnago, Jeanne Marshall, Kristin Sohl, Alicia Brewer Curran, Melissa Mahurin, Melissa Warne-Griggs, WHO CST Team, Pamela Dixon

**Affiliations:** ^1^Department of Psychological Science, University of Texas Rio Grande Valley-Edinburg, Edinburg, TX, United States; ^2^Easterseals Midwest, Columbia, MO, United States; ^3^Department of Child Health, University of Missouri School of Medicine, Columbia, MO, United States; ^4^ECHO Autism Communities, University of Missouri School of Medicine, Columbia, MO, United States; ^5^Missouri Telehealth Network, School of Medicine University of Missouri, Columbia, MO, United States; ^6^Department of Mental Health and Substance Use, World Health Organization, Geneva, Switzerland; ^7^Autism Speaks, Princeton, NJ, United States

**Keywords:** Autism Spectrum Disorder, parent-mediated behavioral intervention, parenting skills training program, ECHO Autism, rural, telehealth

## Abstract

**Background:**

For families with autistic children living in rural areas, limited access to services partly results from a shortage of providers and extensive travel time. Telehealth brings the possibility of implementing alternative delivery modalities of Parent Mediated Interventions (PMIs) with the potential to decrease barriers to accessing services. This study aimed to evaluate the feasibility and acceptability of implementing the World Health Organization-Caregivers Skills Training program (WHO-CST) *via* an online, synchronous group format in rural Missouri.

**Methods:**

We used a mixed methods design to collect qualitative and quantitative data from caregivers and program facilitators at baseline and the end of the program, following the last home visit. Caregivers of 14 autistic children (3–7 years), residents of rural Missouri, completed nine virtual sessions and four virtual home visits.

**Results:**

Four main themes emerged from the focus groups: changes resulting from the WHO-CST, beneficial aspects of the program, advantages and disadvantages of the online format, and challenges to implementing the WHO-CST *via* telehealth. The most liked activity was the demonstration (36%), and the least liked was the practice with other caregivers. From baseline to week 12, communication skills improved in both frequency (*p* < 0.05) and impact (*p* < 0.01), while atypical behaviors decreased (*p* < 0.01). For caregivers' outcomes, only confidence in skills (*p* < 0.05) and parental sense of competence (*p* < 0.05) showed a positive change.

**Conclusion:**

Our results support the feasibility of implementing the WHO-CST program *via* telehealth in a US rural setting. Caregivers found strategies easy to follow, incorporated the program into their family routines, and valued the group meetings that allowed them to connect with other families. A PMI such as the WHO-CST, with cultural and linguistic adaptations and greater accessibility *via* telehealth-plays an essential role in closing the treatment gap and empowering caregivers of autistic children.

## Introduction

Autism Spectrum Disorder (ASD) is a neurodevelopmental disorder characterized by social-communication impairment and repetitive/restrictive patterns of behavior (1). Parents of autistic^1^ children can learn techniques to address the ASD core characteristics, such as promoting communication and social skills, joint attention, positive behaviors, and decreasing restricted and repetitive behaviors in children (3). Parent-Mediated Interventions (PMIs) refer to a group of interventions in which parents are taught strategies typically used by therapists that they can implement with their child in everyday situations. An increasing body of evidence shows PMIs' effectiveness in increasing social interaction and communication, and decreasing atypical behaviors in autistic children (4). In addition, they are frequently used as early intervention protocols for children on the spectrum (3). Common elements of evidence-based PMIs include goal setting, use of behavioral principles, a focus on naturalistic settings and interactions, and systematic evaluation of outcomes (5). The literature indicates that parents of autistic children can learn techniques to promote development and positive behaviors in their children. Previous studies have shown that parents can implement treatment strategies to improve or increase communication skills (6–9), social skills (10) and joint attention (11–13). Besides effectively reducing challenging behaviors and restricted and repetitive behaviors in autistic children (14, 15), PMIs also increase their self-help skills (16). In addition, training in behavioral interventions promotes self-efficacy in parents (17, 18), and parent self-efficacy is associated with positive treatment outcomes for children (11, 19–21). In the U.S., children with an ASD diagnosis between the ages of three and five are also eligible to receive early childhood special education services (22), including speech-language therapy, occupational therapy, and therapy based on the applied behavior analysis (ABA) principles. However, current knowledge about the amounts and types of special education services autistic children have access to is limited (23).

Autism prevalence has doubled in the last decade, increasing service demands and becoming a public health concern (24, 25). Despite the compelling evidence for the efficacy of PMIs, most studies have included parents living in urban areas with more access to services, suggesting that samples do not represent the broader and diverse US population. Consequently, the dissemination of evidence-based interventions is limited among rural communities in the US. Furthermore, there are inequalities in accessing services for autistic individuals living in rural or low-income neighborhoods compared to those living in metropolitan areas (26). Such disparities have been attributed to limited health care resources, shortage of specialized health professionals, and structural factors such as travel distance to service and costs (27). According to the Census Bureau (28), 60 million or 1 in every 5 Americans live in rural areas. Many rural regions have been categorized as shortage areas: geographic areas, populations, and facilities with too few primary care, dental and mental health providers and services [HRSA] (29). As of December 2021, it is estimated that there are 5,999 mental health professional shortage areas in the US, wherein 136 million people reside, with an estimated need of 6,806 mental health practitioners (29). For families with autistic children living in rural areas, limited access to services is partly a function of a shortage of providers and extensive travel time, both for in-home service providers and parents driving to hospitals and clinics that offer services (30). Missouri is among the states with a shortage of health and mental health professionals in the US (31). Children in rural areas are more likely to live in poverty than children in urban settings affecting the health and mental health outcomes of autistic children (32, 33). Therefore, interventions aiming to address such inequalities and social determinants of mental health might differ from families residing in urban or rural settings in the same country (34). Many families with autistic children living in rural areas in the US might not have access to experienced healthcare providers who could make appropriate referrals or offer quality parent-skills training programs. Participation in those services may improve child outcomes, decrease parental stress, and increase parent competency and efficacy (20). Consequently, living in a rural area in the US could be considered a risk factor for diminished access to evidence-based early interventions for autistic children, thus increasing the odds for poorer outcomes in this vulnerable population.

As a response to the global treatment gap for children with developmental disabilities, especially those in low-resource and underserved populations, The World Health Organization (WHO) developed the Caregiver Skills Training (CST) program. WHO-CST is a parent-mediated intervention, freely available and adapted to various settings and levels of care, that aims to decrease the treatment gap for children with developmental disabilities globally, especially those in low-income and underserved settings. The WHO-CST program was developed through extensive stakeholder consultation and an iterative revision, increasing its external validity (35). The WHO-CST program takes (a) a *task-shifting approach*: non-specialists (e.g., social workers and trained community volunteers, caregivers) can deliver this program, (b) a *trans-diagnostic approach:* it does not require a diagnosis to qualify for treatment, and (c) a *common elements approach*: its content focuses on strategies that can benefit a group of caregivers with diverse needs (36, 37). The WHO-CST is delivered *via* nine group sessions and three home visits, providing caregivers with skills that can be used in daily home and play routines. Skills taught in the nine group sessions target social communication, adaptive behavior, and behavior management. The WHO-CST program was developed with the expectation that a community or country will translate and adapt the materials to be culturally relevant without changing the core content. Specific guidance for adaptation is provided with the field-test version of the WHO-CST materials (WHO-CST Team, unpublished). The WHO-CST is currently being adapted and implemented in more than 30 countries worldwide. Outcome evaluations in Ethiopia (38), India (39) and Italy (40) indicate that the WHO-CST is valued as a positive intervention for caregivers and community stakeholders with minimal sociocultural barriers (38–40). The CST's preliminary data from different geographical regions emphasize the adaptation process as essential to ensure its implementation and sustainability. The program was designed to be implemented globally and suitable for low-resource contexts and has shown good acceptability in high-income settings too (40, 41). Thus, the WHO-CST is a sustainable and valid program to implement in the rural US.

Telehealth brings the possibility of implementing alternative delivery modalities of PMIs to decrease barriers to accessing services, such as the limited health care resources and structural factors mentioned above. Telehealth strategies incorporating technology to provide health care services have been explored as a potential solution to the challenges of reaching families in rural settings, resulting in positive caregiver outcomes and satisfaction with services (42, 43). As a result of restrictions associated with the COVID-19 pandemic, caregivers of children with disabilities across geographic settings (i.e., urban, rural) experienced difficulties accessing services. A recent review of telehealth applications for ASD (44) indicates that studies involving PMIs use a variety of approaches to teaching parents strategies to promote child engagement and communication and manage challenging behaviors. Strategies include using video conferencing technology to train parents individually, in groups, and through self-guided websites. Reported outcomes include high levels of parent satisfaction (29, 45, 46), reductions in challenging behaviors (42, 47) and parental stress (48, 49), and improvements in child adaptive functioning (50). Overall, telehealth interventions are well-received by parents and have comparable outcomes to in-person services, providing some components of individual support and coaching (44, 51). The COVID-19 pandemic has exposed the power and usefulness of remote parent support in families with autistic children (39). Empirical data support the efficacy of using PMIs with parents of autistic children, as well as telehealth approaches to deliver PMIs (19, 42, 45, 52–56).

Given the effectiveness of PMIs, the existing barriers to accessing services for families with an autistic child living in rural settings, and the growing body of research supporting the use of telehealth to deliver PMIs, this study aimed to evaluate the feasibility and acceptability of implementing the WHO-CST program *via* an online synchronous group format in rural. Outcomes from this study will support a much larger implementation trial in rural settings.

## Materials and methods

### Study design

The study used a mixed-methods design and collected qualitative and quantitative data from caregivers and program facilitators. A phenomenological framework, as well as a cross sectional survey design, examined the feasibility and acceptability of the WHO-CST in a rural US setting using an online delivery format. In addition, a one-group pretest-posttest with matching design was used to examine if there exists a difference in child and caregivers' outcomes after the program implementation. Data was collected at baseline and at the end of the program, following the last home visit.

Potential participants were referred to research staff at the University of Missouri–Columbia by ECHO Autism clinicians, Missouri Regional Offices and Easterseals Midwest staff. Caregivers who expressed interest in participating in the study were referred to a team member. All participants were contacted by research staff at the University of Missouri–Columbia who provided detailed information about the study. The consent form was verbally reviewed over the telephone for caregivers who wanted to participate. Study data were collected and managed using Research Electronic Data Capture (REDCap) (57) hosted at the University of Missouri–Columbia. REDCap was used to send caregivers a unique link to complete the electronic consent and additional questionnaires.

Following recruitment and consent, participants completed the pre-intervention questionnaires. Each caregiver was assigned to one of three different groups, depending on the time of the scheduled sessions they selected during recruitment. Starting and finish weeks were the same for all groups, but each group offered different days and times for their sessions. Participants attended nine, 90-min group sessions in an online format (conducted through Zoom) and three, 60 to 90-min virtual home visits. One additional 15-min virtual home visit occurred for families after session one to review a goal setting sheet and answer questions the family had regarding their first session.

At the end of the nine group sessions and the four home visits, participants completed the post-intervention questionnaires. Attendance and post session feedback were collected at every encounter. Master Trainers and caregivers participated in separate post-intervention focus groups during December 2020. Group sessions lasted between 40 and 60 min each and were conducted by an experienced independent qualitative researcher who was not a facilitator of any group in the study.

All study procedures were approved by the University of Missouri-Columbia Institutional Review Board, and caregivers, Master Trainers, and Facilitators provided informed consent before collecting study data.

### Participants

Eligibility for the study included caregivers of children between 24 months and 9 years of age diagnosed with ASD. In addition, to be included in the study, caregivers and children were required to (1) be residents of one of Easterseals Midwest's rural catchment areas at the time of the study (i.e., Central, Northeast, or Southeast Missouri), (2) have a reliable internet connection, and (3) access to Zoom through a desktop computer, laptop, or tablet with a video camera embedded or attached, (4) consent to videotaping of virtual home visits, and (5) be fluent in English. There were no additional exclusion criteria.

Caregivers of 18 children completed the initial screening procedures and signed electronic consent forms. Sixteen caregivers completed baseline outcome measures, but only 15 completed the program (one caregiver did not complete post-intervention measures). Table 1 presents the demographic characteristics of the sample. Most caregivers were female (92.9%, *n* = 13), White-non-Hispanic (78.6%, *n* = 11) and had some college education (78.6%, *n* = 11). Half of the sample had received prior training on similar topics (50%, *n* = 7). Children in the study were mostly male (64.3%, *n* = 9), with a mean age of 4.5 years (SD = 1.63). In terms of the history of services, 79 % (*n* = 11) reported having received support from the schools. However, the questionnaire did not inquire about the type of support that was offered. Caregivers also indicated that children had received behavior therapy (57%, *n* = 8), speech–language therapy (71%, *n* = 10), and medication (29%, *n* = 4); but without specification of where that service was provided (school, clinic, or home).

**Table 1:** Demographic characteristics of the participants.

**Characteristics**	***N*** **= 14**
Caregivers demographics	
Gender	
Female	13 (92.9%)
Male	1 (7.1%)
Age, mean ± SD	37.07 ± 5.51
Caregivers education	
High school diploma	1 (7.1%)
Some college	6 (42.9%)
Bachelor's degree	3 (21.4%)
Graduate degree	4 (28.6%)
Ethnicity	
Non-hispanic/non-latino origin	14 (100%)
Race	
White, non-hispanic	11 (78.6%)
African American, non-hispanic	2 (14.3)
White, hispanic	0 (0%)
African American, hispanic	0 (0%)
Not reported	1 (7.1%)
Marital status	
Single	2 (14.3%)
Married	8 (57.1%)
Divorced	3 (21.4%)
Living with romantic partner	1 (7.1%)
Children under 18 at home	
1	1 (7.1%)
2	8 (57.1%)
3	2 (14.3%)
4+	3 (21.4%)
Other children with developmental delays	2 (14.3%)
Relationship with child with developmental delays	
Mother	12 (85.7%)
Father	1 (7.1%)
Grandmother	1 (7.1%)
Had received training on similar topics	7 (50%)
Had received information on this topic	11 (78.6)
Child demographics	
Gender	
Female	5 (35.7%)
Male	9 (64.3%)
Age, mean ± SD	4.53 ± 1.63
History of Services	
School support	11 (79%)
Speech/language therapy	10 (71%)
Behavior therapy	8 (57%)
Medication	4 (29%)

### Intervention

**Figure 1:**
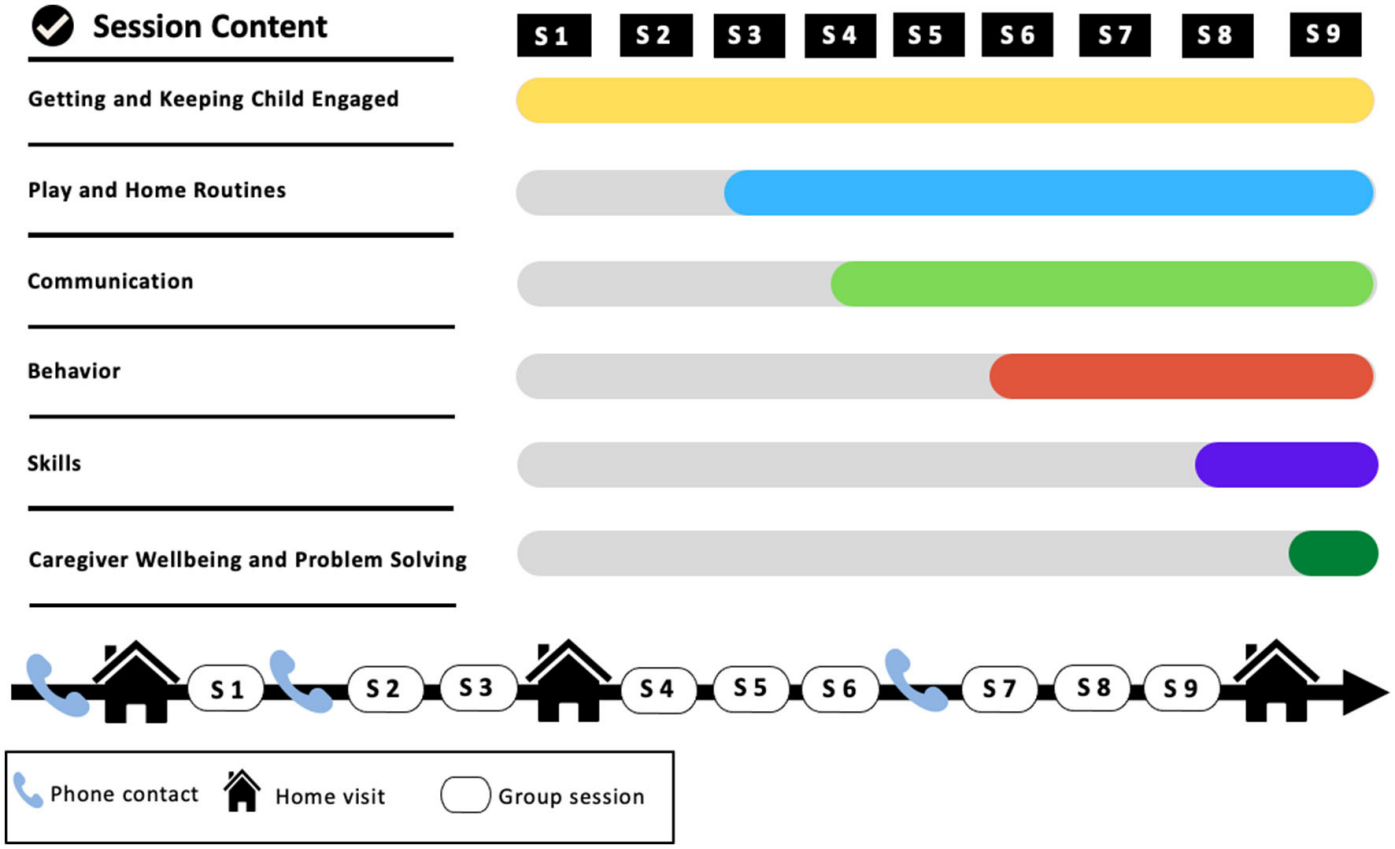
WHO-CST curriculum.

The WHO-CST program was designed to teach caregivers strategies to engage their child in communication and play, to promote adaptive behaviors and learning, and to reduce challenging behavior (35). Its content is based on principles of social learning theory, positive parenting, ABA, and developmental theories. The program consists of a combination of nine group sessions for caregivers and three individual home visits. The content of each of the nine sessions is as follows: (a) introduction and psychoeducation, (b) engaging with the child, (c) helping children share engagement, (d) understanding communication, (e) promoting communication, (f) preventing challenging behavior, (g) responding to challenging behavior, (h) learning new skills, and (i) caregiver problem solving and self-care (Figure 1). For the group sessions, Facilitators implement various techniques, including modeling, role-play, demonstrations, group discussions, and case vignettes. Each session includes homework assignments to encourage caregivers to implement the learned skills in everyday home situations. Before starting the program, Facilitators complete a home visit to define specific goals and targets for each family, explore the presence of additional health problems the child may have, observe the caregiver and child's interactions, inform and engage other caregivers, and answer questions about the program. For the other two home visits occurring halfway through the program and after the last group session, Facilitators focus on coaching the caregiver and providing tailored support, evaluating progress, troubleshooting, and identifying possible additional support needs.

#### Adaptation and pre-pilot testing

A group of stakeholders met to review the materials and assess the need for adaptations for the rural Midwest setting. Stakeholders included four parent trainers with a minimum of 6 years of experience teaching caregivers with autistic children. At this beginning stage, adaptations were mainly linguistic (i.e., changing British spellings to American English) and adapting examples and names to be more culturally representative of the population, consistent with the adaptation guide for the WHO-CST (WHO-CST Team, unpublished). These four parent trainers simultaneously completed a 5-day in-person intensive training with the World Health Organization-Autism Speaks (WHO-AS) team. This training included reviewing the WHO-CST materials, role-playing the sessions, and conducting live practice sessions with mothers and their autistic children. Each Master Trainer met treatment fidelity of at least 80% with the WHO-AS team. Another adaptation to the WHO-CST training of Master Trainers was the supervision component. Typically, Master Trainers send implementation videos of the WHO-WHO-CST model to the WHO-AS trainers and one-on-one feedback is provided. During this pilot, the team adapted the supervision process by leveraging the Extension for Community Healthcare Outcomes (ECHO) model^®^ to aid Master Trainers in achieving fidelity to the WHO-CST model.

ECHO is a model that utilizes video-conferencing technology to provide professionals, such as clinicians, educators, and advocates with the knowledge and guided practice needed to further develop professional expertise. The model has a “hub and spoke” framework that allows spokes, or professionals, to present cases to an interdisciplinary “hub team” of experts. Hub team members mentor and coach spokes to improve spoke knowledge and confidence in their ability to provide best practice care and build a community of practice. The core components of the ECHO model^®^ include a cased-based presentation and a brief didactic. This model has been successfully applied to autism, and the ECHO Autism framework has shown to improve the self-efficacy of community-based clinicians, creating access to high-quality autism care for autistic people and their families in local communities (58).

For this study, the WHO-CST utilized the ECHO Autism framework for supervision and training of Master Trainers (58, 59). WHO-CST teleECHO sessions were led by a hub team of WHO-CST experts that included two WHO-AS trainers and two to four global focal points (i.e., other Master Trainers around the world). Weekly ECHO Autism: WHO-CST teleECHO sessions were hosted by the expert hub team. During the teleECHO sessions, one of the WHO-AS trainers presented a 10-min didactic regarding one of the WHO-CST sessions or primary components. Videos submitted by the Master Trainers were watched by all participants and feedback was given as a group to promote discussion and improved skill application. Fidelity of WHO-CST skills and strategies was evaluated by two clinical psychologists with expertise in implementing the WHO-CST using the WHO-CST Adult/Child Interaction Fidelity scale v1.0 (WHO-CST Team, unpublished). All ECHO Autism teleECHO sessions were completed prior to starting the pre-pilot WHO-CST group.

#### Facilitator training

After completing the pre-pilot testing, the four master trainers conducted a training of four WHO-CST facilitators. The four Master Trainers delivered a 16-h online (i.e., Zoom) training for the facilitators that covered the content of the WHO-CST sessions, group facilitation behaviors, video reviews of the strategies, an introduction to the ECHO Autism framework, and telehealth protocols. Due to COVID-19 protocols in place at that time, Facilitators were not able to practice WHO-CST skills with children in person. As a replacement method, video examples of Master Trainers exhibiting these skills during their fidelity training were used for teaching.

The team leveraged the ECHO Autism-WHO-CST program to provide supervision to Facilitators. ECHO Autism teleECHO sessions occurred once per week while the telehealth WHO-CST program was being delivered, for a total of 14 sessions. During the ECHO Autism teleECHO sessions, one of the Hub Team Master Trainers presented a didactic lesson on the upcoming week's WHO-CST session or Home Visit with the Facilitators. Additionally, Facilitators were scheduled to present two cases. One case presentation focused on the dynamics of the previous week's WHO-CST session, and the second case presentation focused on the experiences the Facilitators had with one caregiver-child dyad that they supported during a home visit. Following rich case discussions generated by the case presentations, the hub team provided Facilitators with feedback and recommendations about how to address any challenges faced during WHO-CST sessions or virtual home visits.

### Measures

During the different stages of the project, caregivers completed a variety of measures to document feasibility and acceptability of the program as well as preliminary efficacy of this delivery method (telehealth) (Table 2), the majority of which were derived from the WHO-CST monitoring-evaluation framework (WHO-CST Team, unpublished).

**Table 2:** Description of the instruments and timeline.

**Purpose**	**Data type**	**Timeline**
*Demographics and* *service history*	Electronic intake questionnaire	Study entry
*Feasibility outcomes*	Attendance	Recorded after each session
	Focus groups	Study exit
	WHO-CST post session feedback form from facilitators	Recorded after each session
	Caregivers knowledge and confidence in skills	Study entry and study exit
*Acceptability outcomes*	WHO-CST post session feedback form from caregivers	Recorded after each session
*Preliminary efficacy* *outcomes*	Autism Treatment and Evaluation Checklist (ATEC)	Study entry and study exit
	Autism Impact Measure (AIM)	
	Brief Family Distress Scale (BFDS)	
	Kessler screening scale for psychological distress	
	Parental stress scale	
	Parenting Sense of Competence (PSOC)	

#### Demographic and service history information

At study entry, caregivers were asked to complete a questionnaire electronically to obtain basic information about the dyad such as the child's gender, age, diagnosis, language spoken at home, ethnicity, and caregiver demographics including gender, relationship to the child, birthdate, and occupation. The caregiver was also asked to report the child's history of medical services and psychological interventions.

#### Feasibility and acceptability outcomes

The following measures were obtained to determine whether the treatment was acceptable and feasible to caregivers and Master Trainers.

(a) **WHO-CST Session Attendance**- Caregivers' attendance at the group and individual sessions was tracked by the program Facilitators.
(b) **Focus Groups–** Focus groups were conducted with WHO-CST Master Trainers and participants. Focus groups followed a guide adapted from questions designed by the CST-WHO developers, consisting of open-ended questions related to expectations, positive experiences, and opportunities for improvement. Additional questions were included to inquire about the online format of the program, and follow-up questions were used to stimulate discussion. All focus groups were conducted in December 2020, and audio recorded *via* Zoom.
(c) **Caregivers Knowledge and Skills Test** (WHO-CST Team, unpublished)– A questionnaire with three sections was given at study entry and study exit to assess caregivers' knowledge and skills related to the WHO-CST content. The first section includes 38 statements about the main skills taught by the WHO-CST. Caregivers are asked to indicate the degree to which they agree with each statement using a 5-point Likert scale (1-Strongly Disagree-5 Strongly Agree). The total score on this subscale ranged from 38 to 190. The second part includes 13 questions about caregivers' confidence in applying some of the skills and knowledge taught by WHO-CST (1-Not at all confident-5 Very Confident), with scores ranging from 13 to 65. The third section is comprised of three vignettes followed by a request a list of three suggestions in how the caregivers in the vignette could respond to the specific situation. For the purposes of this study, sections one and two were included in the analysis. Higher scores are associated with greater knowledge of concepts and strategies taught by the WHO-CST, and confidence in applying those skills with their children.
(d) **Post-session Feedback Form from Facilitators** [adapted from Kasari et al. (11)]-it renders information about acceptability and feasibility of the group sessions according to the Facilitators. Facilitators complete it immediately after each session. Using a 5-point Likert scale, Facilitators will rate the complexity of the content, amount of content for the time of the session, and their perceived preparedness to run the session (feasibility). On the other hand, it requires the Facilitator to rate if the session's content was relevant for caregivers, caregivers' agreement with the ideas presented and their participation and engagement during the sessions (acceptability). At the end it has two open-ended questions about suggestions to improve their preparedness and changes they would make to the session.
(e) **Post session Feedback Form from Caregivers** [adapted from Kasari et al. (11)]- it is a 14-item form that caregivers complete after each session to measure acceptability of group sessions. Using a 5-point Likert scale, caregivers rate the difficulty level of the content (“Did you find this session easy to understand?”), its relevance (“How well do you think the information in this session applies to you and your child?”), usefulness (“How useful will the key messages and tips be to you and your child?”) and alignment with values (“The messages in this session are in conflict with what I believe is good and important”). Another set of questions (multiple choice) inquired about the most and least liked learning activity during the session. With a 3-point Likert scale, 3 questions asked about the length of the whole session, of sharing experiences and discussions, and of practice in pairs (1-Too long 2–Too short 3-Just right). In addition, another section requires the caregivers to rate the usefulness of the WHO-CST tips or strategies that were included in each section. Lastly, there was a space for caregivers to write suggestions to improve the delivery or content of the session (“What could be done differently to improve the session?”).

#### Clinical measures

Several caregiver-report measures were used to examine caregiver and child responses to the WHO-CST program.

(a) **The Autism Treatment and Evaluation Checklist (ATEC)** (60)- This questionnaire was developed to measure changes in response to treatment (60). The ATEC is a one-page 77-item checklist, completed by caregivers, assessing developmental skills and severity of symptoms of developmental delays. It includes four subtests (1) speech/language communication (14 items), (2) sociability (20 items), (3) sensory-cognitive awareness (18 items), and (4) Health/physical behavior. ATEC has been found to be responsive to change in children with autism and shows high internal consistency in the English original and cross-cultural translation (61, 62). Total scores range from 0 to 180, and the higher the score the worse the symptoms.
(b) **The Autism Impact Measure (AIM)** **(****63****)**– The AIM is a caregiver-reported questionnaire assessing autism symptom frequency and impact in children. It was designed specifically for treatment-outcome assessment in children with ASD, focusing on treatment-relevant aspects of symptom presentation and efficient detection of short-term improvement (64). The AIM has 41 parent-rated items, each requiring two corresponding 5-point ratings (frequency and impact). Items reflect either the presence of a maladaptive behavior or the absence of an expected skill.
(c) **Brief Family Distress Scale** (BFDS) (65)– The BFDS is a 10-item parent-report scale designed to indicate the family's level of stress and crisis. A score of one represents perception of positive coping while a score of 10 indicates a marked level of distress where the caregiver perceives the family is currently in crisis. The BFDS has been examined with families of children with ASD. High scores are correlated with problematic coping while low scores are correlated with positive adjustment and coping.
(d) **The Kessler Screening Scale for Psychological Distress (K6)** (66)– The Kessler Psychological Distress Scale (K6+) is a six-item self-report measure of psychological distress intended to be used as a quick tool to assess risk for serious mental illness in the general population, and in this study, it is used in caregivers. It was developed for use in the annual US National Health Interview Survey and National Household Survey on Drug Abuse (66). A cut-point of K6≥13 is the accepted score for serious mental illness (67).
(e) **Parental Stress Scale** (68)– The Parental Stress Scale is a self-report scale that contains 18 items representing pleasure or positive themes of parenthood (emotional benefits, self-enrichment, personal development) and negative components (demands on resources, opportunity costs and restrictions). Respondents are asked to agree or disagree with items in terms of their typical relationship with their child or children and to rate each item on a five-point scale: strongly disagree (1), disagree (2), undecided (3), agree (4), and strongly agree (5). The eight positive items are reverse scored so that possible scores on the scale can range between 18 and 90. Higher scores on the scale indicate greater stress. This scale was designed to assess outcomes of interventions designed to support parenting efficacy of caregivers.
(f) **Parenting Sense of Competence (PSOC)** (69)– The PSOC is a 17-item scale designed to measure parents' satisfaction with parenting and their self-efficacy in the parenting role. Parents indicate their level of agreement with each item by circling a number between 1 (strongly agree) and 6 (strongly disagree). Eight items are reverse scored so that high scores indicate positive parental experience. It has two different subscales: (1) parenting satisfaction (PSOC-S) defined as the person's liking of the parenting role, and (2) parenting efficacy (PSOC-E), defined as the person's perceived competence in the parenting role (69). For this study, we used the Parental Efficacy subscale (PSOC-E).

### Data analysis

#### Statistical analysis

Statistical significance was evaluated at the 0.05 level, and data analyses were performed using IBM SPSS, version 26. Child and caregiver characteristics were calculated for the full sample using means and standard deviations for continuous variables and frequencies and percentages for categorical measures. To evaluate change in outcomes among participants, baseline scores of child and caregivers' outcomes and post intervention scores were compared with paired *t*-tests.

#### Qualitative analysis

Audio recordings of interviews were transcribed using an automated transcription service (Temi.com). To maintain participant privacy, recordings were de-identified before placing them into a secure folder shared with the research team. To ensure accuracy of transcription, the research team compared each transcript to its recording and made any needed edits to match the audio. After each focus group or interview, the focus group facilitator noted important points and lasting impressions from the interview. To inductively derive themes from the data, de-identified transcripts of the audio recordings were used to conduct a content analysis. Two members of the research team conducted the thematic analysis using Excel to organize the data. Although the aim of this study is not to produce grounded theory, we used the technique of constant comparison to identify emerging themes in focus groups and interviews (70). After reviewing these codes, the researchers refined them by consolidating and sorting into broader themes. New codes were added, as needed. Once the analysis was completed, the research team met to develop a summative grid of the emerging themes. Researchers reviewed 273 decision points and found initial agreement of 65% across all decision points. Discussion further clarified code definitions and researchers consulted the original transcripts as needed on areas requiring discussion. Discussion resulted in 100% agreement for the qualitative analysis. The over-coding and review provide evidence that the qualitative analysis and presentation of findings accurately represent the voices of the WHO-CST participants. These processes were aimed at establishing trustworthiness of the data analysis and results.

## Results

### Feasibility and acceptability outcomes

For this pilot study, feasibility outcomes were measured by parental attendance to WHO-CST sessions, attrition, caregiver's knowledge, and confidence in skills, WHO-CST Post Session Feedback form from Facilitators. Acceptability was measured by the WHO-CST Post Session Feedback form from Caregivers and Facilitators, as described in the measures section. Focus groups offered information for both feasibility and acceptability as well as recommendations to improve the program.

#### Feasibility

##### Attendance and attrition

The program included nine group-sessions and three home visits. Attendance rate for the group-sessions was 96.8%, and a 100% for the three home visits. None of the caregivers dropped out, attaining a 0-attrition rate for the online program.

##### Facilitators' ratings of feasibility of delivery of group sessions

Table 3 reports frequencies of ratings in the insufficient (≤2) or excessive (≥4) in the dimensions of complexity of ideas (content of sessions), amount of content for the allotted time, and preparedness to conduct the session according to the Facilitators' perception.

**Table 3:** Facilitators-rated perceived feasibility of delivery of group session.

**Session**	***N***	**Complexity of ideas**				**Amount of content for the time**				**Preparedness to conduct**	
										**the session**	
		***Too simple***		***Too advanced***		***Too little***		***Too much***		***Inadequate***	
		***n***	**%**	***n***	**%**	***n***	**%**	***n***	**%**	***n***	**%**
1	8	1	12.25	1	12.25	2	25	4	50	3	37.5
2	8	0	0	1	12.25	0	0	6	75	1	12.5
3	8	0	0	0	0	0	0	6	75	0	0
4	8	0	0	0	0	1	12.5	7	87.5	0	0
5	8	0	0	0	0	1	12.5	4	50	0	0
6	8	0	0	2	25	0	0	3	37.5	0	0
7	7	1	14.29	0	0	0	0	5	71.4	0	0
8	8	0	0	0	0	0	0	0	0	0	0
9	8	0	0	0	0	0	0	2	25	0	0

##### Caregivers' knowledge and confidence in skills test

As shown in Table 4, caregivers did not exhibit differences in the knowledge pre and posttest (*t* = 0.121, a ≥ 0.05), but they did exhibit differences in confidence scores (*t* = 2.11, a ≤ 0.05). There was no correlation between knowledge gained in the WHO-CST and having participated in previous training or having received similar information in the past.

**Table 4:** Changes in clinical measures: Child and caregivers outcomes.

	**Baseline**		**End of program**		**Baseline to end of program** **mean difference (SE)**	**Paired *t-*test**
	**Mean**	**SD**	**Mean**	**SD**		
***Child outcomes***						
ATEC						
Communication	17.57	5.14	14.14	9.07	3.43 (4.40)	2.92^*^
Sociability	16.54	4.67	14.64	6.91	2.00 (5.97)	1.25
Sensory/Cognitive	19.64	6.63	20.50	7.40	−0.86 (3.48)	−0.92
Health/Physical	24.79	9.10	26.36	11.31	−1.57 (11.63)	−0.506
Total	78.64	12.16	75.64	11.32	3.00 (14.09)	0.80
Autism Impact Measure (AIM)						
*Frequency*						
Communication	19.86	3.98	17.21	4.54	2.64 (1.04)	2.54^*^
Repetitive behavior	22.21	5.95	22.14	6.53	0.07 (5.84)	0.46
Social reciprocity	15.29	4.25	15.43	4.03	−0.14 (3.86)	−0.14
Peer interaction	10.79	3.33	11.00	2.22	−0.21 (2.72)	−0.29
Atypical behavior	16.21	4.02	15.50	4.97	0.71 (4.94)	0.54
Total	116.5	16.25	111.14	15.89	5.36 (5.52)	1.23
*Impact*						
Communication	18.79	5.51	14.31	5.17	4.46 (2.88)	5.59^**^
Repetitive behavior	15.00	5.11	14.69	6.60	0.31 (5.26)	3.49
Social reciprocity	10.86	4.20	11.14	4.55	−0.29 (4.23)	−0.253
Peer interaction	9.54	5.11	8.08	3.57	1.46 (3.48)	1.52
Atypical behavior	18.00	5.45	12.61	5.12	5.38 (5.52)	3.52^**^
Total	95.46	29.09	82.15	30.52	13.31 (20.36)	2.36^*^
***Caregiver outcomes***						
Brief family distress scale	2.86	1.29	2.50	1.34	0.36 (1.15)	1.16
Parental stress scale	62.29	4.85	63.07	4.01	−0.79 (9.36)	−0.58
Kessler-psychological distress	25.36	3.37	24.86	2.38	0.50 (1.52)	0.82
Caregivers knowledge	82.43	3.41	82.29	2.95	0.14 (4.40)	0.12
Caregivers confidence in skills	46.79	9.06	52.07	7.23	−5.28 (9.36)	−2.11^*^
Parental sense of confidence	34.14	5.60	37.29	5.70	−3.14 (5.08)	−2.31^*^

* p < 0.05.

** p < 0.01.

#### Acceptability

##### Caregiver ratings of acceptability of group sessions

In general, caregivers considered the content somewhat easy, somewhat relevant, very useful and not in conflict with their values (Table 5). Only one parent reported that the content of session 6 (Preventing Challenging Behavior, Helping Children Stay Engaged and Regulated) was slightly in conflict with his/her values. Although the caregivers rated the duration of group sessions as “just right,” the specific time allotted for sharing experiences and discussions was considered too short across sections but more frequently for the first three sessions (Table 6).

**Table 5:** Caregivers' rated acceptability of contents of the sessions.

**Session**	***N***	**Difficulty** ^**a**^		**Relevance** ^**b**^		**Usefulness** ^**c**^		**Alignment with values** ^**d**^	
		***n***	**%**	***n***	**%**	***n***	**%**	***n***	**%**
1	13	0	0	1	7.7	0	0	0	0
2	15	0	0	0	0	1	6.7	0	0
3	7	0	0	0	0	0	0	0	0
4	12	0	0	0	0	0	0	0	0
5	12	0	0	0	0	1	8.3	0	0
6	11	0	0	0	0	0	0	1	9.1
7	12	0	0	0	0	0	0	0	0
8	11	0	0	0	0	0	0	0	0
9	8	0	0	0	0	0	0	0	0

a ≤ Somewhat relevant to most participants.

b ≤ A few ideas are not acceptable.

c ≤ Did not express positive or negative opinions about the material.

d ≥ Responded and participated only when prompted.

**Table 6:** Caregivers-rated acceptability of the duration of the different activities of groups sessions.

**Session**	***N***	**Group sessions**				**Sharing experiences/discussion**				**Practice in pairs**			
		**Too long**		**Too short**		**Too long**		**Too short**		**Too long**		**Too short**	
		***n***	**%**	***n***	**%**	***n***	**%**	***n***	**%**	***n***	**%**	***n***	**%**
1	13	1	7.7	0	0	0	0	4	30.7	0	0	2	15.4
2	15	1	6.7	0	0	1	6.7	5	33.3	0	0	2	13.3
3	7	0	0	2		0	0	3	43	0	0	1	14.3
4	12	0	0	0	0	0	0	2	16.7	0	0	0	0
5	12	0	0	0	0	0	0	2	16.7	0	0	0	0
6	11	0	0	0	0	0	0	2	18.2	0	0	1	9.1
7	12	1	8.3	0	0	0	0	0	0	0	0	0	0
8	11	0	0	0	0	0	0	0	0	0	0	0	0
9	8	0	0	0	0	0	0	0	0	0	0	0	0

##### Facilitator ratings of perceived acceptability of group sessions to caregivers

Table 7 shows Facilitators ratings of perceived relevance and acceptability of the sessions' content for the caregivers, agreement with ideas presented during the sessions and caregiver's participation and engagement during the sessions. Frequencies reported are of unsatisfactory ratings (≤ 3).

**Table 7:** Facilitator-rated perceived acceptability of the group sessions to caregivers.

**Session**	***N***	**Relevance** ^**a**^		**Acceptability** ^**b**^		**Agreement** ^**c**^		**Participation** ^**d**^	
		***n***	**%**	***n***	**%**	***n***	**%**	***n***	**%**
1	8	0	0	0	0	1	12.5	3	37.5
2	8	0	0	2	25	3	37.5	5	71.4
3	8	0	0	0	0	0	0	1	12.5
4	8	0	0	0	0	0	0	1	12.5
5	8	0	0	0	0	0	0	2	25
6	8	0	0	0	0	0	0	1	12.5
7	7	0	0	0	0	0	0	2	25
8	8	1	12.5	0	0	0	0	1	12.5
9	8	1	12.5	0	0	1	12.5	3	37.5

a ≤ Somewhat relevant to most participants.

b ≤ A few ideas are not acceptable.

c ≤ Did not express positive or negative opinions about the material.

d ≤ Responded and participated only when prompted.

##### WHO-CST post session feedback form from caregivers

Caregivers' feedback included content's complexity, relevance, usefulness, and alignment with family values. In addition, it measured the parent's preparedness to practice the learned strategies at home. For all the dimensions assessed across the nine sessions, caregivers offered scores equal to or > 3 (neutral), with no scores of 2 or 1 (unsatisfactory cuts off). Most of the caregivers' ratings of content's complexity (73%), relevance (66%), and usefulness (60%) were greater than or equal than 4 (somewhat easy) for all nine sessions. The most liked activity throughout the program was the demonstration (36%), and the least liked was the practice with other caregivers.

##### Focus groups: Feasibility and acceptability

All Master Trainers were interviewed (*n* = 4). Although all caregivers were invited, 10 out of 16 participated in the focus groups. There were between four and six participants in each group, which is consistent with recommended sizes and availability of the participants (71). The thematic analysis of the focus groups with participant caregivers and Master Trainers identified four main themes: (1) Changes resulting from the CST, (2) beneficial aspects of CST, (3) advantages and disadvantages of the online format, and (4) challenges to implementing WHO-CST *via* telehealth (Table 8). Such themes were further qualified according to feasibility, acceptability, and suggestions following the method used by Salomone et al. (40). Excerpts presented are from various participants [caregivers (CST) and Master Trainers (MT)] and in that way present a broad range of experiences and meanings to illustrate the themes and subthemes of this sample.

**Table 8:** Feasibility, acceptability, and suggestions: themes developed from focus groups with caregivers and Master Trainers.

**Domain**	**Themes**			
	*Changes resulting from the WHO-CST*	*Beneficial Aspects of WHO-CST*	*Advantages and disadvantages of the online format*	*Challenges to Implementing WHO-CST *via* Telehealth*
**Acceptability**	Parents increased their confidence in their parenting skills. Changes in children included fewer tantrums, positive reactions to strategies, increased independence for the child, and increased self-expression.	The information is accessible, easy to use, and language was easy to receive. Most useful aspects were visual strategies (i.e., Thermometer), giving the child choices, and breathing exercises. The discussion portion of the group sessions and the Home Visits were the most useful aspects of the program.	Participants thought the online format was convenient. The virtual format made the program accessible to caregivers. Allows participation of caregivers from all over the state.	Too much content to cover in Home Visit 1.
**Feasibility**	Families were engaged and implemented the program into their family routines.	Strategies easy to follow. Learning from and connecting with other families. Some topics were too simple.	Trade-off between community building and accessibility was worth it. Less opportunity to build rapport. Parents seemed to be more focused on getting the content than in sharing with one another. Poor internet connection.	Maintain and practice the learned strategies. Topics or suggestions no applicable to their child. Model working with a child in an online format.
**Suggestions**	Add information that would provide a longer-term perspective; information that would help them know what they might expect down the road. Incorporate a planned follow up.	Update some of the examples to be more relevant to the US context. More coaching time for caregivers. Add information about how to engage schools and how to modify environments for learning–especially IEPS.	Start Facilitators' training in-person and give them a chance to practice in-person with families.	Create a video library as a resource to show an example of the content. Splitting up the first home visit into two visits. Guidance on training Facilitators.

*Changes resulting from the CST*. Both the caregivers and Master Trainers discussed changes that the caregiver or child had made due to the program including changed thinking, behavior, and attitudes. For example, noticing less miscommunication between caregiver and child. All Master Trainers felt that the program had benefited participants and they could see real changes. The families were engaged and implemented the program into their family routines.

*One of the most positive things was just the end result with the families. Once we implemented it with them and got their feedback and saw how comfortable they were with the information and thankful they were probably the most positive thing that we experienced. (MT,2)*

Some caregivers began to feel more confident in their parenting. Others realized they have an active role in communicating with their child and that they can make changes to their own behaviors that could make a difference in the quality of communication with their child. Caregivers adjusted expectations, started using visuals when they hadn't before, and started paying more attention to “the small things.” One parent mentioned shifting their focus from changing behavior to engaging with their child. Changes in children included fewer tantrums, positive reactions to strategies, increased independence for the child, and increased self-expression.

*I've never really paid attention to how much she may have noticed me putting up the groceries or fixing her food or we definitely didn't play games... I'm not sure if she was able to play games, but just doing those simple engagements; it helped her to be more independent, to speak more independently: “Can we go outside now?”, “Can I have a sandwich?” Just by engaging her a little bit more than, “Okay, are you hungry?” And giving her choices definitely would. Giving her choices instead of saying, “Okay, we're going to have lunch.” (CST2,14)*

According to most caregivers, the program would benefit from having information about how to engage schools and how to modify environments for learning–especially IEPs.

*Maybe to give us more information of how to address some issues that you may run into with having your child in the educational environment, how to address a teacher or school. I would have, liked more information about that. I would have liked to maybe have a small message with tips of how to maybe help your child to modify themselves for that type of environment of school when you cannot be there. (CST2,44)*

Master Trainers suggested the incorporation of a planned follow-up, which could be in the form of monthly group check-in meetings or perhaps visits. This was in response to MTs observations that caregivers seemed to “get it” during their second home-visit, but often reverted to previous habits during the third visit. It seemed caregivers would benefit from sustained support to turn learned strategies into lasting changes in interactions with their children.

*Some more home visits or a different structure. I don't know exactly what, but you know, more, face-to-face more coaching, more time doing that. I think our parents didn't, I mean, they didn't want the program to end. …All our families were working families and they got home, and they got dinner started and they jumped on our call and like had a lot of things going on, but they still like wanted to be there and didn't want it to end. So, there's some kind of better fade out maybe… we were like “there -adios”. That's always an uncomfortable transition. So maybe at the end there could be some different types of transition. (MT,43)*

*Beneficial aspects of CST*. Caregivers spoke positively about WHO-CST content, noting the information was accessible, easy to use, and language was easy to receive. Participants felt the program helped them understand and communicate with their children. They stated connecting with other families that are dealing with the same things was important to them and especially appreciated being able to talk to people who can understand what they are experiencing without judgment. This was mentioned by both caregivers as well as Master Trainers:

*Hearing from other families that are having you know the same kind of issues that you are makes you feel less isolated, and you know, just, it, it feels good to make connections with people who can understand what you're going through. (CST1,10)*

Master Trainers also found communication techniques to be the most useful strategy for caregivers. In addition, they mentioned how caregivers took advantage of learning new techniques, or even revisited things that they had seen before but hadn't tried.

*I think... their communication with the parents, the caregivers (was very important); understanding that they have a role in that. It's not just about like their child asking them or telling them, but it's also about how they set the foundation upon which they can do that. Whether it's the way they communicate, the supports, environmental supports they're providing those kinds of things. (MT,38)*

In addition, all the caregivers felt like the discussion portion of the group trainings was the most useful aspect of the program. The discussions helped caregivers think through the strategies they were learning. Caregivers appreciated getting others' feedback (including Facilitators) on the weekly plans they developed, including hearing about others' experiences. They especially liked hearing what worked and didn't work with other people's children. On the other hand, some caregivers felt that some training topics were a little simple, but they understood that people might be coming in with varying levels of education and experience on the issues.

*So, I think that the most useful part of the program for me was the discussions that we had. I felt like the, the lessons were a little bit elementary, and I understand the need for that because, you know, the people that you're working with, aren't always at the same educational level and things, but I personally found the lessons kind of like, okay, this is what we're doing, but the discussion afterward related to those subjects was really good. (CST,39)*

Caregivers frequently mentioned virtual home visits as a key component of the WHO-CST experience and expressed that these visits provided an opportunity to get one-on-one attention and ask questions specific to their child and their needs as a caregiver.

*It gave you an opportunity to have that one-on-one where somebody could help you understand, that (you) might have a question that you don't want to ask in front of a group of people. And so, it made it feel like a safer space in regard to, if you had something that necessarily you didn't want to share...With the larger group. (CST2,35)*

Master Trainers were also largely supportive of the content. They did not suggest removing anything form the program but updating some of the examples to be more relevant to the American context. An example might be changing the images in the participants' booklets.

*I think because of the picture, the illustrations, there's only so many different directions we could take it.... we're not washing our hands in a water basin in the United States. So, like, that's kind of like, “Oh, okay. That's interesting”. I mean, some of the examples we could switch up, some of them we couldn't. And it's not to say it's bad or wrong, but some of those things would be helpful I think, to be updated. (MT,46)*

Master Trainers also made suggestions regarding the training of Facilitators.

*I do wish that there was more guidance on how to properly train the Facilitator. Cause I felt like we were just kind of treading water to figure out what it looks like, reading something there, there wasn't really guidelines on what it should look like, what they need to know, what they don't need to know. So, it was just kind of thrown at us. (MT,31)*

The other recurrent suggestion was to incorporate more coaching to caregivers and maybe more virtual home visits

*So, I think a lot of coaching during that time or more home visits. What I observed was, you know, you had the initial visit where they didn't know anything. You had the midway through visit, and you could see all these changes. And then you have a last home visit. And it was like, all the parents totally forgot what you taught them. It was really interesting. It was like the program's done. And then we came a week or two later and they weren't in it anymore. So, I thought that was really interesting that I did not see it, but when they were in the group, it was phenomenal. So, I think more coaching is what I would like to see. (MT,40)*

*Advantages and disadvantages of the online format*. Participants felt the virtual format made the program accessible to them. They could participate in a comfortable space, not have to travel, or not need to find childcare. Most participants felt like the trade-off between community building and accessibility was worth it.

*I really enjoyed it. I was able to fit it in with all the other services and everything else that we have going on. It was a lot more convenient to schedule. I didn't have to be at a specific location, you know, on top of time travel and all that. It was a lot easier to work into my schedule. (CST1,30)*

*I feel like I was able to fit [it in]. I'm a stay-at-home mom. So, I was able to make every session versus probably having to be like, ‘I can't find a babysitter', especially during these times. So, I think that worked well. (CST2,25)*

Master Trainers indicated that the Zoom format allowed people from all over the state to come together, so it is possible for people to participate regardless of their location relative to program Facilitators.

*I think the advantages is that like our group, we have families from all across the state, you know, so I live up in northern Missouri, and originally that was kind of the recruitment area. And I think we only ended up maybe having one or maybe two out of five families that were actually in that county coverage area. And so, they just kind of picked what day of the week worked best for them. So that was the beauty of the tele-health portion. (MT,30)*

In general participants thought the online format was *convenient*. The only down sides were the lack of human contact, and some tech issues. The main disadvantage discussed by both groups was less opportunity to build rapport.

*If you were actually meeting in person, it would allow you the opportunity to possibly meet somebody with a child with that's the same age and possibly create a friendship out of that. But I mean, that's not even necessarily something that wouldn't happen (online), but I find that when you have a child that you have, you know, different, differently-abled, it's harder to come by those kinds of relationships at times. So that's something that would be positive from that experience, and they can relate. (CST2,28)*

Other issues included technology issues which were mainly problems with internet connections, some awkwardness in interactions such as lulls because people weren't sure who was talking or who might want to go next and dealing with some additional distractions when participating from home.

*Sometimes when it would cut out, it was challenging, but my main thing was on the virtual. I don't even know how the classroom setting would work either, but like pinpointing a specific person to talk because it kind of creates a lot of lull time when everybody's just waiting to see you. And then if you have somebody who's more interested in, you know, communicating everything, you know, it causes other people not to have their perspective looked at and then something that could actually help everybody is missing out on. (CST2,19)*

*Challenges to implementing WHO-CST via telehealth*. At least three caregivers mentioned at some point that there were topics or suggestions that were not applicable to their child, since they thought the suggestion would not work with their children.

*I think sometimes there were some suggestions that I was like, (my son) is never going to do that. Nope. He's not going to cooperate with that. And, you know, I mean, we all know our kids best, so it's worth a try, you know, of course. And then you're like, well, I, I tried it, it did not work and we're going to move on and try something else. Hmm. (CST1,25)*

Participants also found it challenging to maintain and practice the strategies they were taught.

*The only hardest thing was actually finding time to remove all objects out of your area to find time to initiate the engagement for her. That was my still kind of is my biggest problem, finding the time to create engaging time with her. And I'm still kind of looking at her behavior to kind of build and modify some of the messages and tips that were, was given throughout the program. But I think that's still, like my biggest hurdle is finding time to engage and utilize and put the skills to work. (CST2,40)*

Master Trainers mentioned that feedback to Facilitators is not built into how the WHO-CST runs yet. They did not feel this was covered in their Master Trainer training. Most felt coaching Facilitators over Zoom was sufficient, but it was challenging to address ongoing problems. It was also difficult to model working with a child *via* Zoom.

*I had a difficult experience with my Facilitator because I felt like I needed to give her feedback about how she presented and things like that. And that's not really built into the way that WHO-CST is run yet when we're helping to support those Facilitators. So, adding a piece to help Master Trainers do that would be nice to see. (MT,8)*

Although caregivers and Master Trainers considered virtual home visits as one of the most helpful components of the program, Master Trainers felt that the one-first home visit model meant there was a lot for caregivers to digest in one visit. There was not enough time to build rapport, so doing observations felt somewhat awkward. Their suggestion was to focus on program information during the first visit and then do the parenting observation in the second visit. However, caregivers did not mention this as problematic from their point of view.

*[The first visit] was just so much... I think part of that had to do with the recruiting piece because we don't know what they know and they don't know what they don't know, you know? So, it's like trying to figure out what information they have and what they need to expect. And, and that first like home visit, I mean, you have to, you know, we've all been on the receiving end of like coaching that first time you get coaching or someone's watching you and you're trying to, you know, be productive and, you know, effective in what you're doing. And I think families just, they naturally are very hesitant and embarrassed. (MT,25)*

*I think splitting up the first home visit into two visits, I think would have just kind of like set them on a different path and like trajectory, because I feel like they were all, our group was pretty good about communicating with us and they seemed like they were getting the information, but I feel like, like it would have brought it to the next level if we really figured out how to build that rapport. (MT,26)*

Master Trainers also suggested creating a video library as a resource for next telehealth WHO-CST

*In-person, we were able to like model examples and basically role play what it would look like when working with the child. Whereas on Zoom, it wasn't that easy and there also wasn't videos available. So, that would be something I would suggest for telehealth is if they could have a video library ready to go for the next go around that Master Trainers can use to show an example of that, that material that we just went over. (MT,22)*

### Changes in clinical measures

Testing of changes on clinical measures focused on parental knowledge about ASD, parental stress, self-efficacy, and parental reports of child changes as measured by validated instruments.

#### Child outcomes

From baseline to week 12, ATEC communication scores decreased from 17.57 (SD 5.14) from baseline to 14.14 (SD 9.07) at week 12 (*t* = 2.92, *p* < 0.01). Communication scores also improved according to the AIM, in both frequency (*t* = 2.54, *p* < 0.05) and impact (*t* = 5.59, *p* < 0.01). For the frequency communication domain, the scores decreased from 19.86 (SD: 3.98) at baseline to 17.21 (SD: 4.54) at week 12; while for the impact domain they went from 18.79 (SD: 5.51) to 14.31 (SD: 5.17). The AIM impact scores also showed a reduction between baseline and week 12 for atypical behavior (*t* = 3.52, *p* < 0.01) and the total impact score (*t* = 2.36, *p* < 0.05). Atypical behavior impact scores went from 18.00 at baseline to 12.61 at week 12; while the total impact scores decreased from 95.46 (SD 29.09) at baseline to 82.15 (SD: 30.52) at week 12.

#### Caregiver outcomes

Although there was a reduction in all the caregivers' measures, there were statistically significant differences from baseline and week 12 only for the caregiver's confidence in skills (*t* = −2.11, *p* < 0.05) and the parental sense of competence efficacy subscale (*t* = 2.31, *p* < 0.05). Parental sense of competence went from 34.14 (SD: 5.60) at baseline to 37.29 (SD: 5.70) at week 12, indicating an increase in parenting efficacy at the end of the program. Regarding caregivers' confidence in skills, scores went from 46.79 (SD: 9.06) to 52.07 (SD: 7.23) showing an increased confidence in using the learned skills. There were no significant differences for the brief family distress scales, the caregiver's knowledge, or the parental stress scale (Table 4).

## Discussion

This study's primary aim was to evaluate the feasibility and acceptability of implementing the WHO-CST program *via* an online, live group format in rural Missouri. Globally, most research on PMI has been conducted and evaluated in high-income and high-resource settings, leaving out non-urban settings with limited access to services (4). Our results support the feasibility of implementing the WHO-CST program *via* telehealth in a US rural setting. Attendance has been referred to as one of the main barriers to PMI implementation. A zero-attrition rate, 96.8% attendance for the group sessions, and 100% for the four virtual home visits are evidence of the feasibility of implementing the WHO-CST in a telehealth format in a US rural setting. In addition, data collection of outcome measures over 12 weeks was 100%, supporting the feasibility of this format, too. The program was considered feasible by both caregivers and Master Trainers. They reported that the strategies were easy to follow, and that caregivers were engaged and incorporated the program into their family routines. Caregivers also valued the group meetings that allowed them to learn from and connect with other families.

The COVID-19 pandemic enlarged health disparities for ASD services in low-resourced areas (39). Telehealth has been rapidly growing, and the COVID-19 pandemic increased its use and acceptance and showed us that remote PMI was a possibility (47, 72). Even before the pandemic, some research showed that PMI *via* telehealth is an effective delivery modality for addressing core symptoms and challenging behaviors in autistic children (39). The benefits are seen in many aspects, including scheduling, costs, and better use of resources. Other PMI implementation studies have shown that participation barriers are parents' time and childcare for non-autistic children and transportation (38). For our research, the online format diminished such hurdles as parents expressed.

The telehealth format made the WHO-CST program accessible to rural caregivers, allowing participation of caregivers from across the state. According to reports from the caregivers, the drawback of this format is that it offered less opportunity to build rapport among the participants. In addition, technical issues like internet connectivity, screen freeze, and timing lags were seen as challenges; however, caregivers stated that the benefits outweighed the challenges. Telehealth could be a feasible alternative for those families who experience the most common barriers to accessing services. The remote model allowed families from remote rural places to join the sessions, making WHO-CST available where previously there were no services. Sengupta et al. (39) commented on the need to offer options for the in-home visits in settings where cultural or contextual barriers might hinder the physical home visits. Our results support the use of telehealth for such purposes.

Caregivers rated the program content and its activities as comprehensible, relevant to them, and aligned with their values across sessions and home visits, which supports its acceptability in the context of rural American Mid-West. Results from different measures show that the demonstration was the most liked activity, whereas practice with pairs was the least liked one. These are similar findings to those from Salomone et al. (40) in Italy's WHO-CST field trial. For this study, one of the adaptations was to use prerecorded video for the demonstration activities, so Facilitators could pause, rewind, or replay it to illustrate or explain the content. The practice in pairs activity was challenging to complete in this virtual format. Caregivers rated the time devoted for the pair's activities as too short. Thus, the combination of the remote effect and not enough time to complete the activity could have resulted in its dislike. Salomone et al. (40) reported similar findings in their in-person trial. Being an essential part of the training and a good learning strategy for non-specialists, role-playing activities need to be revised to find different formats to present them in a more engaging and timely fashion. Caregivers reported that one of the main strengths of the WHO-CST was the opportunity to share with other caregivers that experience the same concerns, making it clear that the interaction is not the barrier here but the way the activity is presented. Outside COVID-19 circumstances, remote WHO-CST would offer caregivers more options to access services. WHO-CST might constitute an added option in service providers' portfolios that could eventually reduce operational costs by pairing the remote format (online) with the WHO-CST curriculum, thus increasing the probability for attendance and positive caregiver and child outcomes. Facilitator ratings of the feasibility of remote delivery of group sessions showed that sessions 2 (Keeping Children Engaged), 3 (Helping Children Share Engagement in Play and Home Routines), 4 (Understanding Communication), and 7 (Teaching Alternatives to Challenging Behavior) had too much content to present in the time allotted for each of the sessions. However, caregivers rated the level of difficulty of such sessions as adequate or somewhat easy, and the length of the sessions as 'just right. The delivery method (online) might have placed a burden on Facilitators, making them feel they did not have enough time to present all the content. However, one of the adaptations made for this study was to reduce the length of the sessions to avoid participants' fatigue. During the focus groups, Facilitators recommended using videos to demonstrate the content or present them as examples. Such a modification might ease the load of Facilitators to deliver the content in the planned time. Likewise, the WHO-CST implementation in northern Italy showed that some sessions were also considered as too packed with information. Such results suggest the need to revise those sessions for future implementations. Also, alternative instructional strategies like videos or more guided role-playing might diminish caregivers and Facilitators burden.

The other feasibility measure was knowledge of the skills related to WHO-CST content. Caregivers did not show any difference in pre-WHO-CST knowledge and post CST. However, the sense of confidence significantly improved, implying that the WHO-CST program might have an influence on learning how to use the knowledge, and applying the learned knowledge. Knowledge might not have been new for caregivers but practicing the concepts during group sessions or virtual home visits might have given caregivers confidence in using the skills. The characteristics of our sample (42.9% had some college, 21.4% had a college degree, 78.6% had previously received information on similar topics) might have impacted the results. The social determinants of mental health framework proposes that the circumstances in which people live, and work shape their health outcomes (73). Social determinants include SES, education, physical environment, access to healthcare (74). The high baseline knowledge scores may also indicate that the instrument's questions were too easy for the participants. The questionnaire was designed to be administered in low resourced/low-and middle-income countries; hence, it might need to be adapted to reflect the characteristics of a more educated sample of caregivers. Even if most caregivers had already participated in similar training or educational sessions on autism and parenting, they found the program's content beneficial.

During the focus groups, caregivers and Facilitators considered virtual home visits an essential element of the program. In that way, we might see the impact of live coaching on caregivers, which is an added value of the WHO-CST compared with self-paced PMI (75). In addition, naturalistic interventions like CST, which embed learning and practice opportunities into the child's daily routines, increase skill generalization (19). Facilitators perceived that the information gathered in the first home visit was too much for the caregivers to process. They suggested splitting it in two, so the first home visit could be devoted to discussing program information and the second home visit could focus on observing the caregiver-child interaction. The adapted curriculum added a 15-min home visit for families after session 1 to review a goal-setting sheet and answer questions the family had regarding their first session. In that way, the Facilitators enhanced the opportunities to interact with caregivers, clarify the goals for each family, and answer questions the participants might have had. Facilitators indicated the benefit of including a planned follow-up, which could be in the form of monthly group check-in meetings or perhaps visits. They supported the suggestion by stating that caregivers might understand the content during the second visit, but that learning is not present during the third visit. They conclude that caregivers would benefit from sustained support to turn learned strategies into lasting changes in interactions with their children. Results from the Indian WHO-CST trial also suggested the need for ongoing support for parents to build their competency in implementing strategies in the form of booster sessions post-implementation (39).

Parents have expressed high levels of satisfaction with therapist-assisted telehealth interventions. Such interventions have been associated with acquiring knowledge of behavioral interventions strategies from such programs (76). In other studies, parents reported reduced stress and increased self-efficacy after participating in a telehealth PMI (19). Iadarola et al. (20) provided outcome data on caregiver stress and parenting sense of confidence. Parents in the treatment arm of the study reported greater improvement on the parenting sense of confidence (effect size 0.34) than parents in the parent education program (effect size 0.34). Conversely, Bradshaw et al. (77) showed that parents in the parent education program reported a significant reduction in parenting stress and increased parenting sense of competence. These results, aligned with ours, might suggest that participation in group parent training could be a valuable tool that could increase self-efficacy and reduce parental stress.

Regarding participants' suggestions to improve the program, caregivers proposed having sessions with content related to engaging their child's school and longer-term perspectives on autism to help them prepare for the future. In addition, Facilitators indicated that having a video library would be beneficial when presenting new content or demonstrating/teaching new skills to caregivers. Both Facilitators and caregivers suggested updating some of the examples to be more relevant to the American context. Specifically, they commented that session two content relative to witchcraft or demon possessions was not relevant for the type of families in this geographic location, mainly white American Midwest. According to the adaptation guidance, this information should be used to further adapt the program to the context in Missouri. Compared to the Salomone et al. (40, 41) study, the only published field trial in a high-income country, caregivers, and Facilitators voiced similar concerns with the caregivers' stories. For example, one of the Facilitators pointed out that people do not use a basin to wash their hands in the US, while a caregiver pointed out the absence of fathers in most of the stories. Such comments highlight the relevance and need for an adaptation process aligned with cultural practices and values in the community in which the WHO-CST will be implemented.

It is possible that caregiver stories may not require changes in other settings in which the story contents are a well-recognized and culturally relevant tool (38, 40). For the WHO-CST implementation in rural Missouri, the central adaptations were linguistic: changing British spelling to American English, as well as examples and names to have culturally and linguistically valid materials. Overall, caregivers identified implementation barriers related to maintenance and practice of the learned strategies and the use of some contents that did not apply to their child's developmental level. Facilitators referred to challenges in terms of the amount of content for the first home visit and how to model some WHO-CST strategies to the caregivers *via* an online format. Besides the provided examples, caregivers and facilitators deemed the adapted WHO-CST materials acceptable and relevant for the American rural Midwest.

Although not the primary aim of the study, analysis of clinical measures from our sample suggests that the efficacy of the WHO-CST program delivered *via* telehealth is promising. However, due to the design, the non-probabilistic sampling process, and other limitations of the study, these results need to be considered as preliminary. The significant results aligned with the primary outcomes of the program: provide caregivers with strategies to support their children's development by engaging children in everyday activities and applying strategies to support the development of the child's communication skills and reduce challenging behavior. Results showed a significant reduction in the AIM atypical behavior impact scores but not in the frequency, suggesting that the stigma experienced by parents comes from atypical behaviors (78). Using the skills learned during the WHO-CST program, parents might have learned how to redirect, manage, and perceive the behaviors. In addition, there was a significant decrease in the AIM Total impact scores, but not for the frequency. This change may represent how the WHO-CST might have impacted the way caregivers interpret behaviors. Children might still exhibit the same behavior, but caregivers are given a different meaning to such behaviors due to their participation in the WHO-CST program. Nevertheless, it is important to mention that some of the participants had received services, limiting our ability to make inferences about the factors impacting the clinical outcomes.

There were no significant changes for the social interaction measures. For families and autistic individuals' global events like the pandemic could add distress to an already complex scenario that might have impacted children's opportunities for social interaction. Additionally, the interruption of face-to-face schooling, leisure activities, and reduced access to services represented a disruption of routine, which might partly be responsible for the absence of changes in the social interaction area (79, 80). In summary, interventions deployed to control the spread of the COVID-19 pandemic such as social distancing and the use of face masks, may conflict with interventions aimed to improve the wellbeing of autistic children (81), such as the WHO-CST program. WHO-CST is a naturalistic behavioral intervention, and as such, implementation takes place during naturally occurring home and play routines requiring high levels of clinical judgment. In consequence, training of non-specialists might need more practice and coaching than more directive and structured interventions (82–84). Other studies have found that administrative support, the interactive nature of the training, and the compatibility of the training model with Facilitators' current practices facilitate the training process (54). Although due to the COVID-19 pandemic, our Facilitators received remote training; all Master Trainers and Facilitators had extensive training and experience with children with neurodevelopmental disorders and behavioral interventions. Their current activities included parent training sessions and in-home consultations. WHO-CST was designed to be implemented by non-specialists, but on our site, it was delivered by behavioral health workers with extensive experience in child development and autism; as was the case of Italy (40, 41) and India (39). Therefore, our results are limited to settings that use Facilitators with similar educational and experience backgrounds.

The results of this study need to be interpreted in light of its limitations. First, there was a small sample and no control group. However, there were four dyads of Master Trainer-Facilitator implementing the program independent of each other. Second, we did not have independent raters of children and caregivers' behaviors, which could have biased the results. Third, we used a mixture of qualitative and standardized quantitative self-report measures to evaluate changes in clinical outcomes rather than performing an independent objective examination of knowledge and skills. However, there is no consensus on what measures to use for the range of potentially relevant outcomes to evaluate intervention effectiveness for autistic children (85). Finally, all families were paid for participation in all assessments, impacting the attrition rate and data collection rates. In terms of Facilitator training, due to COVID-19, Facilitators completed their entire training *via* Zoom, preventing them from obtaining hands-on training with families, including the opportunity to see interactions with families. Master trainers agreed that some in-person training would be beneficial. However, the results from this study suggest that remote training for Facilitators might also be an option with some adaptations to the programmed practice. Other studies have found that telehealth is an effective way for training non-specialists in delivering PMIs, and also for providing supervision and coaching (42).

Albeit these limitations, this study has several strengths. It is the first implementation of a telehealth PMI conducted with non-specialists in a rural US setting; and could be generalizable to similar rural settings. Results from this study-an adapted parent-mediated intervention-could impact public policy by offering a scalable and sustainable program to bridge the gap between research and community implementation. Furthermore, this study innovates by (1) including a unique sampling framework to represent caregivers from an underserved rural setting, (2) using the ECHO Autism model to prepare WHO-CST group Facilitators in a cost effective and efficient manner while allowing for iterative guidance with WHO-CST implementation, and (3) applying a mixed methods design to inform the process. Results would serve as a baseline for future autism studies and service interventions with underserved rural families in other rural US areas. Our mixed-methods approach using focus groups ensures the adaptations made to the WHO-CST are relevant to rural families of autistic children.

Glasgow and Emmons (86) advocate using practical trials with both quantitative and qualitative methods to assess multiple outcomes relevant to community implementation since delivery of professional services is complex and more so in a naturalistic setting such as the presented trial. These preliminary results using a mix-methods design do not allow us to draw conclusions about the efficacy of the CST-online format. Nevertheless, these findings provide information about the feasibility of implementing the WHO-CST program *via* telehealth in an underserved rural setting, contributing to the global field trials of the program, and serving as preliminary data for a larger randomized control trial (RCT) to explore its efficacy. According to Bearss (42), remote PMI is an effective delivery modality for core symptoms and challenging behaviors in autistic children. In a systematic review and metanalysis, Deb et al. (87) urged experts to standardize a PMI for autistic children and carry out a large-scale RCT to assess its clinical and economic effectiveness. WHO-CST might be that tool since it: (a) uses evidence-based procedures, (b) is open access, (c) can be administered by non-specialists, (d) requires cultural and linguistic adaptation, (e) uses a community based-participatory framework increasing the odds for sustainability and scalability, (f) does not require a diagnosis, decreasing time for accessing services, (g) is a low dose-low intensity program, (i) could be used in both low-and-middle income and high income countries, and (j) has promising evidence of its effectiveness when used *via* telehealth.

For implementing a naturalistic intervention, such as WHO-CST, in a rural setting, several factors unrelated to the PMI need to be identified to increase the success of its implementation. Among such factors, engagement of community stakeholders and partnership with a specialized autism center is essential to offer support to Facilitators and participants from rural areas. In our case, both Easterseals Midwest and the ECHO Autism at the University of Missouri-Columbia provided the knowledge, supervision, and administrative capabilities to conduct the trial. In addition, ECHO Autism clinicians provided a healthcare network that allowed for the referral of families to the program. Lastly, a reliable internet connection for caregivers and service providers is needed, as well as engaging caregivers with technical knowledge to access the sessions' links and materials.

High-income-countries have many low-resource contexts, such as rural areas and the health professional shortage areas in the US (29), in which families have limited access to services. Although the source and degree of disparities might differ, low-resourced communities in high-income-countries share similar characteristics of low-and-middle-income-countries in terms of barriers accessing timely and evidence-based interventions and shortage of trained professionals to identify and treat children with neurodevelopmental conditions (88). In these instances, a PMI-such as the WHO-CST with cultural and linguistic adaptations and greater accessibility *via* telehealth-plays an essential role by closing the treatment gap and empowering caregivers of autistic children.

## Data availability statement

The raw data supporting the conclusions of this article will be made available by the authors, without undue reservation.

## Ethics statement

All study procedures were approved by the University of Missouri-Columbia Institutional Review Board. The patients/participants provided their written informed consent to participate in this study.

## Author contributions

AC, CM-N, KS, JM, MT, and PD contributed to conception and design of the study. AC, JM, MT, and MM collected the data. CM-N organized the database, performed the statistical analysis, and wrote the first draft of the manuscript. AC, MT, MW-G, and PD wrote sections of the manuscript. All authors contributed to manuscript revision, read, and approved the submitted version.

## Funding

This project received funding from Autism Speaks.

## Conflict of interest

The authors declare that the research was conducted in the absence of any commercial or financial relationships that could be construed as a potential conflict of interest.

## Publisher's note

All claims expressed in this article are solely those of the authors and do not necessarily represent those of their affiliated organizations, or those of the publisher, the editors and the reviewers. Any product that may be evaluated in this article, or claim that may be made by its manufacturer, is not guaranteed or endorsed by the publisher.

## Footnotes

^1^We are aware of the diverse opinions regarding the terminology used to refer to individuals on the spectrum. We have elected to use identity-first instead of person-first language following the suggestions to avoid ableist language (2).
